# Supplementary material for: Decentralized study of COVID Vaccine Antibody Response (STOPCoV): Results of a participant satisfaction survey
Source: PLOS Digit Health. 2023 May 9;2(5):e0000242. doi: 10.1371/journal.pdig.0000242 (PMC10168576; doi:10.1371/journal.pdig.0000242)
Supplement: Attachment — Submitted filename: review_plosone.docx [file pdig.0000242.s001.docx]

Dear STOPCoV study authors,

I congratulate you on your brilliant work during the COVID-19 Pandemic. The completion of this study brings a great contribution to evidence-based science. Satisfaction studies are important tools for designing public health policies. Below are my considerations regarding the writing of the manuscript.

Abstract: Remove this information: (The research was built on a REDCap database, reviewed and approved by the University Health Network Research Ethics Board)

Introduction:

Your introduction does not provide clear and contextualized information about the problem addressed.

Suggested organization for the Introduction:

- Present a brief conceptualization of the COVID-19 Pandemic, relationship of worldwide and Canadian epidemiological data.

- Summarize the changes that have occurred in society and the challenges that have been overcome.

- Bring a link from these challenges to the new practices of evidence-based science (Information presented in the first paragraph of your introduction).

- The second paragraph I recommend restructuring by contextualizing the information already available in the medical literature (Studies already published by your own research group).

- Present the objectives of the study clearly and objectively.

Methods: Dear authors, although you have referenced studies that present the same methods, I believe this information should be present in this study as:

- Study design: all information regarding the type of study, ethical approval, and study registration information.

- Sample selection: characterize the sample studied and the number of participants included.

- Present data on the geospatial demographics of where this study was conducted so that readers of PLOS Digital Health can situate themselves.

- Describe how the construction of the satisfaction evaluation matrix was conducted. Highlight in this session why satisfaction was assessed.

- Information Collection: Detail how the researchers obtained the responses (example: number of emails sent, time to trigger emails... ).

Data Analysis: Describe how the data was evaluated and the tests used.

Questions/Considerations about the information described in the method.

The three individuals selected to conduct the pilot study were individuals of what age range? Would they represent the age profile of the study? Was there any reliability analysis of the questionnaire applied to these individuals?

With regard to consent, were the individuals interviewed given an online form explaining the risks and benefits of the study?

Results: Table 1 of participant demographics.

- I suggest dividing the participants into three categories (Male, Female, and Non-Binary). I believe the authors have grouped the information for a leaner statistical analysis, however, this grouping cannot be done because they are different categories.

- I suggest reviewing the concepts between race and ethnicity to classify the participants. If it was self-reported by the participants, this should be described in the methodology of the study.

- BMI b : I suggest removing this line because it does not provide any information.

Questions/ Considerations about the results presented.

Throughout the text, information about age and sex has not been described. To make it easier to read, I suggest adding these values to the table.

Congratulations again for conducting this work.

Sincerely,
