# Supplementary material for: Decentralized study of COVID Vaccine Antibody Response (STOPCoV): Results of a participant satisfaction survey
Source: PLOS Digit Health. 2023 May 9;2(5):e0000242. doi: 10.1371/journal.pdig.0000242 (PMC10168576; doi:10.1371/journal.pdig.0000242)
Supplement: Attachment — Submitted filename: Response to reviewers plos.docx [file pdig.0000242.s002.docx]

Monday, January 30, 2023

We thank you for the careful review of our manuscript and suggestions which have helped to strength it. We trust that with the changes we made as listed below that it will now be suitable for publication in your journal. We address the comments in order and indicate our response in red.

Dear STOPCoV study authors,

I congratulate you on your brilliant work during the COVID-19 Pandemic. The completion of this study brings a great contribution to evidence-based science. Satisfaction studies are important tools for designing public health policies. Below are my considerations regarding the writing of the manuscript.

Thanks for your kind comments, we are very proud of this study and have been amazed at the ongoing interest and participation of our study subjects.

Abstract: Remove this information: (The research was built on a REDCap database, reviewed and approved by the University Health Network Research Ethics Board)- done

Introduction:

Your introduction does not provide clear and contextualized information about the problem addressed. –Suggested organization for the Introduction:

- Present a brief conceptualization of the COVID-19 Pandemic, relationship of worldwide and Canadian epidemiological data. we have added a paragraph about the status of the pandemic as we intiated the study and why this was important

- Summarize the changes that have occurred in society and the challenges that have been overcome.- done

- Bring a link from these challenges to the new practices of evidence-based science (Information presented in the first paragraph of your introduction). – done

- The second paragraph I recommend restructuring by contextualizing the information already available in the medical literature (Studies already published by your own research group).- done

- Present the objectives of the study clearly and objectively. – done

Methods: Dear authors, although you have referenced studies that present the same methods, I believe this information should be present in this study as: added all these details about the main study and the rapid antigen test substudy

- Study design: all information regarding the type of study, ethical approval, and study registration information.- done

- Sample selection: characterize the sample studied and the number of participants included.- done

- Present data on the geospatial demographics of where this study was conducted so that readers of PLOS Digital Health can situate themselves.- done

- Describe how the construction of the satisfaction evaluation matrix was conducted. – done

Highlight in this session why satisfaction was assessed.- done

- Information Collection: Detail how the researchers obtained the responses (example: number of emails sent, time to trigger emails... ).- this was presented but reworded for clarity

Data Analysis: Describe how the data was evaluated and the tests used.- this has been added to the methods section

Questions/Considerations about the information described in the method.

The three individuals selected to conduct the pilot study were individuals of what age range? – added Would they represent the age profile of the study? – yes , added Was there any reliability analysis of the questionnaire applied to these individuals?- no

With regard to consent, were the individuals interviewed given an online form explaining the risks and benefits of the study? – yes this was done and added to text

Results: Table 1 of participant demographics.

- I suggest dividing the participants into three categories (Male, Female, and Non-Binary). – we understand but because there were only three in the nonbinary category we did not want to break any confidentiality- the numbers are so small they cannot be compared separately

I believe the authors have grouped the information for a leaner statistical analysis, however, this grouping cannot be done because they are different categories.

- I suggest reviewing the concepts between race and ethnicity to classify the participants. If it was self-reported by the participants, this should be described in the methodology of the study. – it was self-reported and we note this

- BMI b : I suggest removing this line because it does not provide any information.- we removed the data on BMI as suggested

Questions/ Considerations about the results presented.

Throughout the text, information about age and sex has not been described. To make it easier to read, I suggest adding these values to the table. – the tables do separate the responses by age category

Congratulations again for conducting this work.- we thank you and look forward to this work being published and shared with others

We also provide reponses to the following:

Reviewer #2: In the manuscript entitled “Decentralized study of COVID Vaccine Antibody Response (STOPCoV): Results of a participant satisfaction survey.”, the Authors performed a sub-study from the main STOPCoV study to determine the satisfaction in decentralized study and compare the effect of age (70+ vs 30-50 years old).

The Authors major findings show a high overall satisfaction and moderate problems regarding procedure and blood collection.

This research addresses an important topic regarding the use of decentralized and web-based clinical trial, raised from SARS-CoV-2. However, there are some major points that need to be addressed:

Again, we thank you for the strong support of our study

Major Points:

- The study lacks of a formal analysis of Likert scale to compare age groups responses. Please provide statistical analysis and include the main results in Abstract, Results and discuss them in the Discussion sections.

We have completed this analysis and included the p values in the tables and provided them in the results and discussion

- The dataset is highly unbalanced towards older population (21% 30-50 years old). This evidence should be discussed to improve participation of younger age in future studies.

We a prior purposely over-recruited this study in the older cohort- we indicate this in the text

Minor Points:

- A part of the discussion should cover the pros and cons of decentralized studies in respect to on-site research studies.- we have included this in the context of our study

If any further information or clarity required please feel free to contact me.

Sharon Walmsley, C.M, MD, FRCPC

sharon.walmsley @uhn.ca
