# Supplementary material for: Decentralized study of COVID Vaccine Antibody Response (STOPCoV): Results of a participant satisfaction survey
Source: PLOS Digit Health. 2023 May 9;2(5):e0000242. doi: 10.1371/journal.pdig.0000242 (PMC10168576; doi:10.1371/journal.pdig.0000242)
Supplement: Attachment — Submitted filename: StopCov Survey Response to R2 - March 13 2023.docx [file pdig.0000242.s003.docx]

**Response to Reviewers/Editor:**

PDIG-D-22-00252R1
Decentralized study of COVID Vaccine Antibody Response (STOPCoV): Results of a participant satisfaction survey.
PLOS Digital Health

Dear Dr. Walmsley,

Thank you for submitting your manuscript to PLOS Digital Health. After careful consideration, we feel that it has merit but does not fully meet PLOS Digital Health's publication criteria as it currently stands. Therefore, we invite you to submit a revised version of the manuscript that addresses the points raised during the review process.

Please submit your revised manuscript within 30 days Mar 30 2023 11:59PM. If you will need more time than this to complete your revisions, please reply to this message or contact the journal office at [digitalhealth@plos.org](mailto:digitalhealth@plos.org). When you're ready to submit your revision, log on to [https://urldefense.com/v3/__https://www.editorialmanager.com/pdig/__;!!CjcC7IQ!N1iUewQ0AMI9sHc7yWh5XDrzueHrs3YDkyMl66uCKBfGp2FmV4iZ_cwYdHJXarfvdaoaiAVhxeBFn3-x2W_s](https://urldefense.com/v3/__https:/www.editorialmanager.com/pdig/__;!!CjcC7IQ!N1iUewQ0AMI9sHc7yWh5XDrzueHrs3YDkyMl66uCKBfGp2FmV4iZ_cwYdHJXarfvdaoaiAVhxeBFn3-x2W_s)$ [editorialmanager[.]com] and select the 'Submissions Needing Revision' folder to locate your manuscript file.

We have submitted within this time frame.

Please include the following items when submitting your revised manuscript:
* A rebuttal letter that responds to each point raised by the editor and reviewer(s). You should upload this letter as a separate file labeled 'Response to Reviewers'.
* A marked-up copy of your manuscript that highlights changes made to the original version. You should upload this as a separate file labeled 'Revised Manuscript with Track Changes'.
* An unmarked version of your revised paper without tracked changes. You should upload this as a separate file labeled 'Manuscript'.
If you would like to make changes to your financial disclosure, please include your updated statement in your cover letter. Guidelines for resubmitting your figure files are available below the reviewer comments at the end of this letter.

All included

We look forward to receiving your revised manuscript.

Kind regards,

Shlomo Berkovsky
Section Editor
PLOS Digital Health

Journal Requirements:

1. Please review your reference list to ensure that it is complete and correct. If you have cited papers that have been retracted, please include the rationale for doing so in the manuscript text, or remove these references and replace them with relevant current references. Any changes to the reference list should be mentioned in the rebuttal letter that accompanies your revised manuscript. If you need to cite a retracted article, indicate the article's retracted status in the References list and also include a citation and full reference for the retraction notice.

We updated our reference list with our last revision- no further changes

2. Please send a completed 'Competing Interests' statement, including any COIs declared by your co-authors. If you have no competing interests to declare, please state "The authors have declared that no competing interests exist". Otherwise please declare all competing interests beginning with the statement "I have read the journal's policy and the authors of this manuscript have the following competing interests:"

We asked for clarification and received this response: Thank you for your email, and apologies for any unclear instructions. Regarding the COI, you do not need to add this statement to the manuscript, but rather when you resubmit we ask that you please update the statement within the system by adding the leading phrase "I have read the journal's policy and the authors of this manuscript have the following competing interests:"

We have updated the statement in the system as advised

3. Please ensure that Funding Information and Financial Disclosure Statement are matched.

We asked for clarification and received this response: For the funding disclosure, you also do not need to include this in the manuscript. I believe the operator flagged this statement due to the possibility that the statement may be missing a relevant grant/application number for the Public Health Agency of Canada, whose contribution has "CITF ID: 087-VS" in the grant number field in the funding information section of the submission form, as seen below.

There is no further information to add.

Additional Editor Comments (if provided):

Minor revisions/additions suggested by one of the reviewers. Please address these to get the paper to a publishable form.

[Note: HTML markup is below. Please do not edit.]

Reviewers' comments:

Reviewer's Responses to Questions

<font color="black"><b>Comments to the Author</b><br /><br />1. If the authors have adequately addressed your comments raised in a previous round of review and you feel that this manuscript is now acceptable for publication, you may indicate that here to bypass the "Comments to the Author" section, enter your conflict of interest statement in the "Confidential to Editor" section, and submit your "Accept" recommendation.</font>

Reviewer #1: All comments have been addressed

Reviewer #2: All comments have been addressed

--------------------

<font color="black">2. Does this manuscript meet PLOS Digital Health's <a href="[https://urldefense.com/v3/__https://journals.plos.org/digitalhealth/s/journal-information*loc-criteria-for-publication__;Iw!!CjcC7IQ!N1iUewQ0AMI9sHc7yWh5XDrzueHrs3YDkyMl66uCKBfGp2FmV4iZ_cwYdHJXarfvdaoaiAVhxeBFnyeQTBgd](https://urldefense.com/v3/__https:/journals.plos.org/digitalhealth/s/journal-information*loc-criteria-for-publication__;Iw!!CjcC7IQ!N1iUewQ0AMI9sHc7yWh5XDrzueHrs3YDkyMl66uCKBfGp2FmV4iZ_cwYdHJXarfvdaoaiAVhxeBFnyeQTBgd)$ [journals[.]plos[.]org]" target="_blank">publication criteria</a>? Is the manuscript technically sound, and do the data support the conclusions? The manuscript must describe methodologically and ethically rigorous research with conclusions that are appropriately drawn based on the data presented.</font>

Reviewer #1: Yes

Reviewer #2: Yes

--------------------

<font color="black">3. Has the statistical analysis been performed appropriately and rigorously?</font>

Reviewer #1: Yes

Reviewer #2: Yes

--------------------

<font color = "black">4. Have the authors made all data underlying the findings in their manuscript fully available (please refer to the Data Availability Statement at the start of the manuscript PDF file)?<br><br> The <a href="[https://urldefense.com/v3/__https://journals.plos.org/digitalhealth/s/data-availability__;!!CjcC7IQ!N1iUewQ0AMI9sHc7yWh5XDrzueHrs3YDkyMl66uCKBfGp2FmV4iZ_cwYdHJXarfvdaoaiAVhxeBFn-6Vj57b](https://urldefense.com/v3/__https:/journals.plos.org/digitalhealth/s/data-availability__;!!CjcC7IQ!N1iUewQ0AMI9sHc7yWh5XDrzueHrs3YDkyMl66uCKBfGp2FmV4iZ_cwYdHJXarfvdaoaiAVhxeBFn-6Vj57b)$ [journals[.]plos[.]org]" target="_blank">PLOS Data policy</a> requires authors to make all data underlying the findings described in their manuscript fully available without restriction, with rare exception. The data should be provided as part of the manuscript or its supporting information, or deposited to a public repository. For example, in addition to summary statistics, the data points behind means, medians and variance measures should be available. If there are restrictions on publicly sharing data-e.g. participant privacy or use of data from a third party-those must be specified.</font>

Reviewer #1: Yes

Reviewer #2: Yes

--------------------

<font color = "black">5. Is the manuscript presented in an intelligible fashion and written in standard English?<br/><br/>PLOS Digital Health does not copyedit accepted manuscripts, so the language in submitted articles must be clear, correct, and unambiguous. Any typographical or grammatical errors should be corrected at revision, so please note any specific errors here.</font>

Reviewer #1: Yes

Reviewer #2: Yes

--------------------

<font color = "black">6. Review Comments to the Author<br/><br/>Please use the space provided to explain your answers to the questions above. You may also include additional comments for the author, including concerns about dual publication, research ethics, or publication ethics. (Please upload your review as an attachment if it exceeds 20,000 characters)</font>

Reviewer #1: Dear STOPCoV study authors,

Congratulations on the significant improvement of your manuscript. Here are some considerations regarding the methodology and results presented.

1- Present the demographic characteristics of the region studied, since PLOS Digital Health is a journal of international reach. I strongly believe that this information should be present in its methodology.

We have added the following paragraph

**Study Region**: Participants were recruited from across the province of Ontario, Canada. According to Census Canada 2021, there are 14, 223, 942 persons living in Ontario, with a total area of 15.2/km2, and a density of 1 million persons/km2. Approximately 2/3 are in urban areas with Toronto representing the largest metropolitan area, with a population of 6.6M. Of the Ontario population, 95% are English speaking, 34% identify as a visible minority and 5.5% as Black. Of the Ontario population, 12% are men and 14% women between the ages of 30-50, and 4.9% men and 5.7% women between the ages of 70 and 85 years.

2- Since this is a satisfaction study, we believe that the stratification of subjects (Male, Female, and non-binary) is fundamental to understanding the demographic delineation of the population. I understand that from a statistical point of view the ideal would be to group the information. However, in epidemiological studies the richness of detail must be maintained in the analysis. Therefore, I recommend stratifying the information.

We asked for clarification and received this response: It is not necessary to perform regression model. I suggest creating three categories: male, female and non-binary. I believe that this stratification will help us obtain more accurate and relevant information for the investigation on the gender of the population studied.

For each table, we have separated the columns in each age category by gender. P values compare the two age categories which was our original hypothesis and then by gender within each cohort. As there were only 3 individuals that reported gender as non-binary we included them with females in order to maintain confidentiality. For ease of reading, the changes to the tables are highlighted in yellow in the tracked version.

3- Add in Table 01 information regarding the self-declaration of race of the subjects included, since this is not described in the methodology or in the descriptive results.

This is in the methodology and added in the table.

Reviewer #2: I have carefully read the new version of the article "Decentralized study of COVID Vaccine Antibody Response (STOPCoV): Results of a participant satisfaction survey". The Authors made several improvements, especially in the statistical part. All my concerns have been properly answered.

Thank you
--------------------

<font color = "black">7. PLOS authors have the option to publish the peer review history of their article (<a href="[https://urldefense.com/v3/__https://journals.plos.org/digitalhealth/s/editorial-and-peer-review-process*loc-peer-review-history__;Iw!!CjcC7IQ!N1iUewQ0AMI9sHc7yWh5XDrzueHrs3YDkyMl66uCKBfGp2FmV4iZ_cwYdHJXarfvdaoaiAVhxeBFn9xEWsRw](https://urldefense.com/v3/__https:/journals.plos.org/digitalhealth/s/editorial-and-peer-review-process*loc-peer-review-history__;Iw!!CjcC7IQ!N1iUewQ0AMI9sHc7yWh5XDrzueHrs3YDkyMl66uCKBfGp2FmV4iZ_cwYdHJXarfvdaoaiAVhxeBFn9xEWsRw)$ [journals[.]plos[.]org]" target="_blank">what does this mean?</a>). If published, this will include your full peer review and any attached files.<br><br>

<b>Do you want your identity to be public for this peer review?</b> If you choose "no", your identity will remain anonymous but your review may still be made public.<br><br>

For information about this choice, including consent withdrawal, please see our <a href="[https://urldefense.com/v3/__https://www.plos.org/privacy-policy__;!!CjcC7IQ!N1iUewQ0AMI9sHc7yWh5XDrzueHrs3YDkyMl66uCKBfGp2FmV4iZ_cwYdHJXarfvdaoaiAVhxeBFn7ZxnMF8](https://urldefense.com/v3/__https:/www.plos.org/privacy-policy__;!!CjcC7IQ!N1iUewQ0AMI9sHc7yWh5XDrzueHrs3YDkyMl66uCKBfGp2FmV4iZ_cwYdHJXarfvdaoaiAVhxeBFn7ZxnMF8)$ [plos[.]org]" target="_blank">Privacy Policy</a>.</font>

Reviewer #1: No

Reviewer #2: No
